# Pharmacogenomic Identification of c-Myc/Max-Regulated Genes Associated with Cytotoxicity of Artesunate towards Human Colon, Ovarian and Lung Cancer Cell Lines

**DOI:** 10.3390/molecules15042886

**Published:** 2010-04-22

**Authors:** Serkan Sertel, Tolga Eichhorn, Christian H. Simon, Peter K. Plinkert, Steven W. Johnson, Thomas Efferth

**Affiliations:** 1 Department of Otorhinolaryngology, Head & Neck Surgery, University of Heidelberg, Im Neuenheimer Feld 400, 69120 Heidelberg, Germany; 2 German Cancer Research Center, Pharmaceutical Biology (C015), Im Neuenheimer Feld 280, 69120 Heidelberg, Germany; 3 Department of Pharmaceutical Biology, Institute of Pharmacy and Biochemistry, University of Mainz, Staudingerweg 5, 55099 Mainz, Germany; 4 Department of Pharmacology, University of Pennsylvania Cancer Center, Philadelphia, PA 19104-6160, USA

**Keywords:** Artesunate (ART), clinical oncology, chemotherapeutic resistance, microarray, expression profiling, pathway analysis

## Abstract

Development of novel therapy strategies is one of the major pressing topics of clinical oncology to overcome drug resistance of tumors. Artesunate (ART) is an anti-malarial drug, which also exerts profound cytotoxic activity towards cancer cells. We applied a gene-hunting approach using microarray-based transcriptome-wide mRNA expression profiling and COMPARE analyses. We identified a set of genes, whose expression was associated either with high IC_50_ values or low IC_50_ values for ART. Therefore, these genes may function as resistance or sensitivity factors for response of tumor cells towards ART. This viewpoint is conceivable for genes involved in ribosomal activity, drug transport, cellular antioxidant defense, apoptosis, cell proliferation, cell cycle progression *etc*. An investigation of underlying signal transduction by pathway analysis suggested a role of the signaling pathways related to tumor necrosis factor (TNF) and the tumor suppressor p53. On the other hand, there were genes without obvious functional link to cellular response to ART, such as genes involved in the survival of cochlear outer and inner hair cells *etc*. We proved the hypothesis that ART influences the activity of transcription factors regulating downstream genes involved or not involved in response of cancer cells towards ART. This would explain the identification of genes with and without obvious relation to the cytotoxic activity of ART by microarray and COMPARE analyses. By analysis of the binding motifs for the transcription factors c-Myc and Max, we indeed found that 53 of 56 genes contained one or more binding sites for c-Myc/Max upstream of the gene-location. We conclude that c-Myc and Max-mediated transcriptional control of gene expression might contribute to the therapeutic effects of ART in cancer cells, but may also confer unwanted side effects by affecting therapy-unrelated genes.

## Abbreviations

ABC : = ATP-binding cassette transporter
ACT : = artemisinin-based combination therapy
ART : = artesunate
CYP : = cytochrome P450
DEPC : = diethylpyrocarbonat
DMSO : = dimethylsulfoxide
EGFR : = Epidermal Growth Factor Receptor
iNOS : = inducible nitric oxide synthase
IPA : = Ingenuity Pathway Analysis
LPS : = lipopolysaccharide
MAX : = Myc-associated factor x
MDR : = multidrug resistance
MTT : = 3-(4,5-dimethyl-thizol-2-yl)-2,5-diphenyltetrazolium bromide
NCI : = National Cancer Institute
NF-κB : = nuclear factor 'kappa-light-chain-enhancer' of activated B-cells
NSCLC : = non-small cell lung cancer
PCR : = polymerase chain reaction
PDK : = pyruvate dehydrogenase kinase
Pgp : = P-glycoprotein
PI3K : = phosphatidylinositol 3'-kinase
PK : = protein kinase
PKC : = protein kinase C
ROS : = reactive oxygen species
qRT-PCR : = quantitative real-time PCR
SDS : = sodium dodecyl sulphate
SSC : = standard saline citrate
TCM : = traditional Chinese medicine
TGF-β1 : = transforming growth factor-beta 1
TNF : = tumor necrosis factor
WHO : = World Health Organization

## 1. Introduction

*Artemisia annua* L. (Chinese: qīnghāo), also known as sweet annie, sweet sagewort or armoise annuelle, is used in traditional Chinese medicine (TCM) for the treatment of fever and chills [1]. In 1972, Prof. Tu Youyou (Chinese Academy of Traditional Chinese Medicine, Beijing, China), identiﬁed artemisinin (qīnghāosu) as the active anti-malarial constituent of *Artemisia annua* L. [2,3]. Artemisinin is a sesquiterpene lactone with an internal peroxide bridge essential for its activity towards *Plasmodium falciparum* and *Plasmodium vivax* [1,4]. In fact, the World Health Organization (WHO) has officially recommended artemisinin and its derivatives for the treatment of malaria, particularly as a part of combination therapies with other anti-malarial drugs (artemisinin-based combination therapies, ACTs). 

In the past dozen of years, we have systematically analyzed medicinal plants used in TCM for phytochemicals with cytotoxic activity towards cancer cells [5,6,7,8,9] Among a huge panel of natural products, we found that the artemisinin and its derivative artesunate (ART) also reveal profound anti-cancer activity *in vitro* and *in vivo* [4,6,7,8,10,11,12,13,14,15]. So far, their mechanisms of anti-cancer action have not completely been understood. 

In the present investigation, we used microarray technology in order to disclose all genes involved at the transcriptional level. We subjected this expression profile to a signaling pathway analysis. Furthermore, we performed a transcription factor analysis, which indicated a possible role of c-Myc and Max as transcriptional regulators for downstream genes determining the response of cancer cells towards ART.

## 2. Results

### 2.1. Cytotoxicity of cell lines

**Figure 1:**
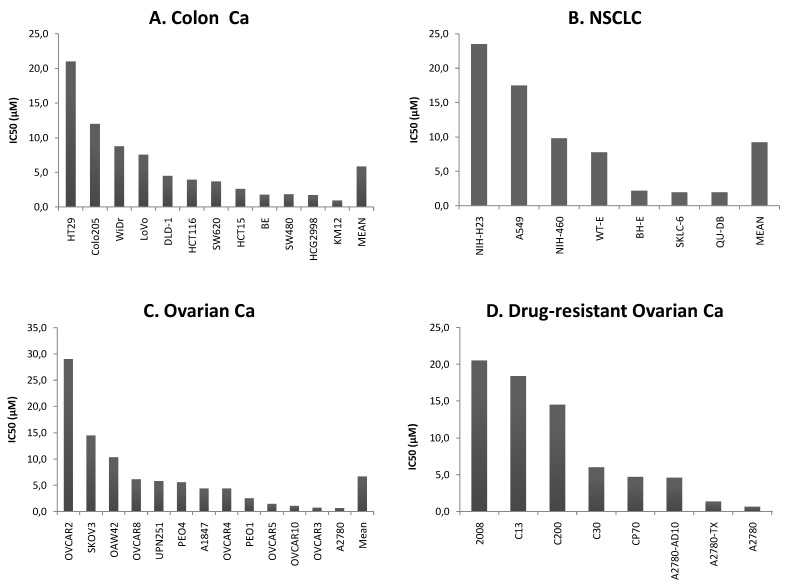
Ranked order of IC_50_ values for ART in 39 human cell lines of three different anatomical locations. (A) Colon cancer cell lines, (B) non-small cell lung cancer (NSCLC) cell lines, (C) ovarian cancer cell lines, and (D) sensitive ovarian cell lines and sublines resistant to cisplatin, adriamycin, or paclitaxel.

The mean 50% inhibition concentration (IC_50_) for ART in colon cancer cell lines was 5.9 ± 5.8 μM (Figure 1A), in non-small cell lung cancer 9.2 ± 8.5 μM (Figure 1B), and in ovarian cancer cell lines 6.7 ± 7.8 μM (Figure 1C). To investigate the activity of ART in drug-resistant cell lines, ovarian cancer cells selected for resistance towards cisplatin, adriamycin, or paclitaxel were used (Figure 1D). While all cisplatin-resistant sublines were similar or more sensitive towards ART than the parental 2008 cell line, adriamycin- or paclitaxel-resistant A2780 cells were cross-resistant towards ART as compared to their drug-sensitive counterpart (Figure 1D).

### 2.2. Microarray hybridization

A pharmacogenomic approach was applied to explore novel molecular determinants of sensitivity and resistance to ART. We determined the transcriptome-wide mRNA expression of 39 tumor cell lines and correlated the expression data with the IC_50_ values for ART. This represents a hypothesis-generating approach, which allows the identification of novel putative molecular determinants of cellular response towards ART. We performed COMPARE analyses of the IC_50_ values for ART and the microarray-based mRNA expression levels. First, we performed a standard COMPARE analysis, in which cell lines that were most inhibited by ART (lowest IC_50_ values) were correlated with the lowest mRNA expression levels of genes. These genes may be considered as possible candidate genes, which determine cellular resistance to ART. Afterwards, a reverse COMPARE analysis was performed: the most inhibited cell lines were correlated with the highest gene expression levels, indicating possible drug sensitivity genes. Only correlations with a correlation coefficient of R > 0.3 (standard COMPARE) or R > -0.3 (reverse COMPARE) were considered (Table 1). 

Among the genes identified by this approach were genes from diverse functional groups such as structural constituents of ribosomes (*RPL29*), ATP-binding cassette (ABC) transporters (*ABCC3*), kinases (*PRKCSH, ITPK1, IKBKG*, *DDR2*), cellular antioxidant defense and carcinogenesis (*ATOX1*), organization of actin cytoskeleton (*RRAS*), carcinogenesis (*SMAD3*, *WNT7A*), cell adhesion and growth of malignant cells (*ST8SIA1*), apoptosis and cell proliferation (*CSE1L*), cell cycle progression and differentiation (*S100A10*) and metastasis (*HMGA1*, *RPSA*).

**Table 1:** Genes determining sensitivity or resistance towards ART in the panel of 39 cell lines as identified by microarray mRNA expression profiling and COMPARE analysis.

RESISTANCE GENES				
Gene	UNIQID	Pearson	Name	Function
*SLC30A1*	R31110	0.552	Human hbc647 mRNA sequence	Involved in zinc transport out of the cell.
*GFAP*	AA069414	0.552	Glial fibrillary acidic protein	Class-III intermediate filament
*BDKRB2*	AA194043	0.513	Bradykinin receptor B2	Receptor for bradykinin
*AKR1C3*	AA916325	0.511	Aldo-keto reductase family 1, member C3	Member of the aldo/keto reductase superfamily
*SNAP23*	N32278	0.472	Synaptosomal-associated protein, 23kD	Regulator of membrane fusion machinery and transport vesicle docking
*KYNU*	H87471	0.471	Kynureninase (L-kynurenine hydrolase)	Cleaves L-kynurenine and L-3-hydroxykynurenine
*AKR1C1*	R93124	0.453	Aldo-keto reductase family 1, member C1	Member of the aldo/keto reductase superfamily
*ITPK1*	AA463931	0.452	Inositol 1,3,4-triphosphate 5/6 kinase	Phosphorylates various inositol polyphosphates
not specified	H44051	0.443	not specified	Member of the keratin family
*ALDH3A1*	AA069024	0.435	Aldehyde dehydrogenase 3 family, member A1	ALDH oxidizes various aldehydes to the corresponding acids.
*GYS2*	N52282	0.432	Glycogen synthase 2 (liver)	Transfers the glycosyl residue from UDP-Glc to the non-reducing end of alpha-1,4-glucan
*KRT4*	AA629189	0.425	Keratin 4	Member of the keratin gene family
*CD24*	H59916	0.423	CD24 antigen (small cell lung carcinoma)	Signal transducer; modulates B-cell activation
*SMAD3*	W72201	0.423	Similar to mothers against decapentaplegic homolog 3	Transcriptional modulator; plays a role in carcinogenesis
*CREB3L3*	AI952482	0.415	Sirtuin 6	Glycosyl transferase activity
*SLC12A7*	AA427732	0.402	Solute carrier 12 (potassium/chloride transporters)	Important for the survival of cochlear outer and inner hair cells
*SLC22A5*	AI933276	0.398	Solute carrier 22 (organic cation transporter)	Involved in the active cellular uptake of carnitine.
*FETUB*	AA705308	0.396	Fetuin B	Member of the fetuin family
*TRAM1*	H15707	0.366	Translocating chain-associating membrane protein	Translocation of secretory proteins across the ER membrane
*WNT7A*	AI885451	0.365	Wingless-type MMTV integration site family	Involved in oncogenesis
*DCDC2*	AA127741	0.360	Doublecortin domain containing 2	Enhances microtubule polymerization
*ERRFI1*	AA400258	0.359	Gene 33/Mig-6	Involved in cell signaling and cell stress
*ABCC3*	AA429895	0.357	ATP-binding cassette, sub-family C (CFTR/MRP)	Member of the superfamily of ABC-Transporters
*IKBKG*	R56102	0.357	Homo sapiens cDNA FLJ20586 fis, clone KAT09466	Regulatory subunit of the inhibitor of kappaB kinase (IKK)
*S100A10*	AA444051	0.354	S100 calcium-binding protein A10	Regulation of cellular processes such as cell cycle progression and differentiation
*GSR*	AA777289	0.352	Glutathione reductase	Maintains high levels of reduced glutathione in the cytosol
*ST8SIA1*	AA169183	0.349	Sialyltransferase 8 A	Cell adhesion and growth of cultured malignant cells
*CUL5*	AA086475	0.337	Cullin 5	May form a cell surface vasopressin receptor
*SCAP*	R54823	0.335	Srebp cleavage-activating protein	Regulates sterol biosynthesis
*AKR1B1*	AA701963	0.330	Aldo-keto reductase family 1, member B1	Member of the aldo/keto reductase superfamily
*ATP1B1*	AA598814	0.329	ATPase, Na+/K+ transporting, beta 1 polypeptide	Catalyzes ATP hydrolysis and ion exchange across plasma membranes
*VCAN*	AA101875	0.326	Chondroitin sulfate proteoglycan 2 (versican)	May play a role in intercellular signaling, cell mobility and differentiation
*TRIM21*	N45131	0.309	Sjögren syndrome antigen A1, tripartite motif-containing 21	Ribonucleoprotein particle which binds DNA, RNA, protein and zinc
*GLRX*	AA291163	0.307	Glutaredoxin (thioltransferase)	Reduces low molecular weight disulfides and proteins
*SLC23A1*	AI934925	0.306	Solute carrier family 23 (nucleobase transporters)	This gene encodes one of the Vitamin C transporters
**SENSITIVITY GENES**				
**Gene**	**UNIQID**	**Pearson**	**Name**	**Function**
*RPL29*	AW073449	-0.300	Ribosomal protein L29	Cytoplasmic ribosomal protein of the 60S subunit
*PSMB5*	AA864479	-0.304	Proteasome (prosome, macropain) subunit, β type	May catalyze basal processing of intracellular antigens
*DDR2*	AA243749	-0.308	Discoidin domain receptor family, member 2	Tyrosine kinase receptor mediating fibroblast migration and proliferation
*ASNS*	AA894927	-0.309	Asparagine synthetase	Synthesis of asparagine
*LDHB*	AI969670	-0.310	Lactate dehydrogenase B	Oxidoreductase activity
*RAD23A*	H99170	-0.310	RAD23 homolog A (S. cerevisiae)	Molecular calcium binding chaperone
*CSE1L*	N69204	-0.312	Chromosome segregation 1 (yeast homolog)-like	May play a role in apoptosis and cell proliferation
*INSIG1*	H59620	-0.314	Insulin induced gene 1	May play a role in growth and differentiation of tissues involved in metabolic control
*PABPCP5*	AA486531	-0.315	Poly(A)-binding protein, cytoplasmic 1	May be involved in translationally coupled mRNA turnover
*ATOX1*	AA418694	-0.315	Antioxidant protein 1 (yeast) homolog 1	May play a role in carcinogenesis
*PRKCSH*	AA496810	-0.316	Protein kinase C substrate 80K-H	Acidic phospho-protein known to be a substrate for protein kinase C
	AA878561	-0.319	Ubiquitin A-52 residue ribosomal protein product	Regulation of gene expression
*ISG15*	AA406020	-0.319	Interferon-stimulated protein, 15 kDa	May regulate proteins involved in the release of prostaglandin F2-alpha (PGF)
*EEF2*	R43766	-0.322	Eukaryotic translation elongation factor 2	Essential factor for protein synthesis
	R37276	-0.322	Eukaryotic translation initiation factor 4 gamma, 1	Recognition of the mRNA cap
*PCDH17*	AA969048	-0.326	Protocadherin 17	May play a role for cell-cell connections in the brain
*PHB2*	AA464567	-0.328	B-cell associated protein	Functions as an estrogen receptor
*TIMM17A*	AA708446	-0.335	Translocase of inner mitochondrial membrane 17	Translocation of transit proteins across mitochondrial membrane
*RRAS*	AI368184	-0.337	Related RAS viral (r-ras) oncogene homolog	Regulates the organization of the actin cytoskeleton
*SYNCRIP*	AA186327	-0.338	Synaptotagmin binding, cytoplasmic RNA binding protein	Involved in mRNA processing
	AA448261	-0.345	High-mobility group protein isoforms I and Y	Gene transcription, integration of retroviruses into chromosomes and metastasis
*RPL18A*	W81118	-0.356	EST, similar to human 60S Ribosomal Protein L18A	unknown
*RPS10*	AI611010	-0.357	Ribosomal protein S10	Catalyzes protein synthesis. Variable expression of this gene in colorectal cancers
*UBB*	AW078798	-0.358	Ubiquitin B	Regulation of gene expression
*RPSA*	AA629897	-0.367	Laminin receptor 1 (67kD, ribosomal protein SA)	Up-regulation in cancer cells associated with invasion and metastasis
*PRMT1*	N55480	-0.381	HMT1 (hnRNP methyltransferase)-like 2	Arginine methyltransferase
*LMAN1*	AA446103	-0.386	Lectin, mannose-binding, 1	Type I integral membrane protein
*YBX1*	AA599175	-0.418	Nuclease sensitive element binding protein 1	May play a role in DNA repair

### 2.3. Signaling pathway profiling

**Figure 2:**
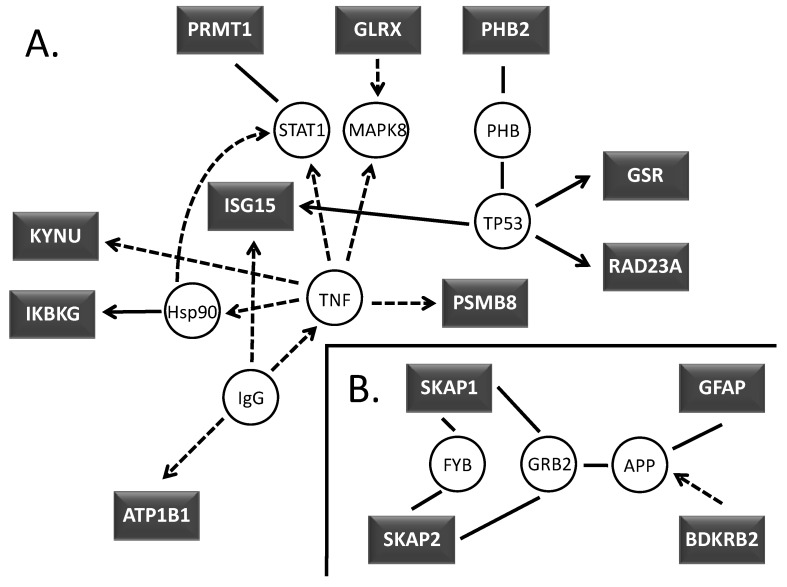
Network view of associated proteins created within Ingenuity Pathway Analysis software for visualizing molecular interrelationships. **(A)** TNF-α and TP53, **(B)** SKAP and GFAP associated proteins. Coloring is based on the expression values that were uploaded with the dataset. Gray indicates the molecule was part of the dataset. Lines connecting molecules indicate molecular relationships. Dashed lines indicate indirect interactions; solid lines indicate direct interactions. The style of the arrows indicates specific molecular relationships and the directionality of the interaction.

As a next step, we employed a signaling pathway analysis to better understand the biological consequences of ART treatment. The genes identified by microarray and COMPARE analyses were subjected to Ingenuity Pathway Analysis (version 6.5). Two networks were found to be significant in that they contained more of the identified genes than expected by chance. The first network is associated with cell morphology, antigen presentation and cell-mediated immune response (Figure 2A). The second network is associated with nervous system development and function, and cellular assembly and organization (Figure 2B). 

### 2.4. Transcription factor downstream gene analyses

In a previous investigation, we found a correlation between c-Myc expression and sensitivity towards ART [16], indicating that transcriptional regulation by c-Myc might play a role for mediating the cytotoxic effects of ART. By means of the ConSite program (http://www.phylofoot.org/consite/) for detection of transcription factor binding sites we now analyzed, whether the genes found in the present microarray analysis contained c-Myc binding motifs in their gene promoter sequences. Out of 56 genes, one gene has twelve, two genes have eleven, three genes nine, one gene eight, four genes seven, seven genes six, six genes five, five genes four, twelve genes three, five genes two potential binding sites, and seven genes have one potential binding site for c-Myc (Table 2). Remarkably, only three out of 56 gene promoters did not contain c-Myc binding motifs, indicating that transcriptional regulation by c-Myc might be an important determinant of cellular response towards ART.

**Table 2:** Binding sequences of the transcription factor c-Myc upstream of genes identified by microarray mRNA expression profiling (**+**, 5’-3’ strand; **-**, 3’-5’ strand).

Gene	Number of potential binding sites	Position (Score)	Strand
***SLC30A1***	3	-2645 (7,989), -910 (8,955), -909 (9,341)	+, +, -
***GFAP***	2	-4972 (8,199), -161 (9,706)	+, -
***BDKRB2***	7	-4885 (9,431), -4884 (8,449), -3439 (8,229), -2826 (8,977), -2825 (8,229), -1493 (8,820), -1466 (8,372)	+, -, +, +, -, +, +
***AKR1C3***	4	-3486 (9,956), -3182 (8,252), -1029 (7,972), -71 (9,667)	+, -, -, -
***SNAP23***	3	-4877 (8,036), -857 (8,449), -856 (9,431)	-, +, -
***KYNU***	**No binding sites detected**		
***AKR1C1***	1	-4606 (8,039)	+
***ITPK1***	1	-2885 (8,890)	-
***GYS2***	1	-4280 (8,960)	-
***KRT4***	12	-4540 (9,810), -4539 (8,393), -3905 (8,504), -3532 (9,421), -3531 (10,337), -3442 (8,372), -2264 (9,431), -2263 (8,449), -1942 (10,480), -1941 (13,638), -1016 (8,841), -165 (9,118)	+, +, -, +, -, +, +, -, +, -, -, +
***CD24***	3	-2956 (9,104), -2955 (9,909), -2906 (8,055)	+, -, -
***SMAD3***	5	-3843 (7,989), -3135 (9,044), -2558 (8,372), -418 (9,341), -417 (9,341)	-, -, -, +, -
***CREB3L3***	9	-4911 (10,153), -3674 (8,186), -32229 (8,162), -2802 (8,449), -2801 (9,431), -2073 (10,396), -1327 (9,585), -485 (9,431), -484 (8,449)	-, +, +, +, -, +, +, +, -
***SLC12A7***	11	-4608 (10,961), -4607 (9,095), -2657 (8,252), -2584 (10,769), -2204 (8,504), -1970 (9,134), -1862 (8,820), -1209 (9,238), -1016 (8,229), -61 (10,468), -60 (9,869)	+, -, -, -, -, +, -, -, -, +, -
***SLC22A5***	**No binding sites detected**		
***FETUB***	6	-3871 (8,074), -1418 (8,464), -804 (10,572), -803 (8,441), -418 (9,706), -69 (9,983)	-, +, +, -, +, -
***TRAM1***	3	-4348 (8,890), -4347 (9,552), -3118 (8,515)	+, -, +
***WNT7A***	3	-4745 (9,706), -2862 (9,491), -2664 (8,960)	-, +, +
***DCDC2***	1	-2660 (8,259)	+
***ERRFI1***	2	-4501 (8,372), -4141 (8,761)	+, +
***ABCC3***	5	-4552 (8,120), -4326 (7,989), -4097 (8,229), -4064 (9,044), -4063 (8,229)	+, +, +, +, -
***IKBKG***	3	-952 (9,431), -951 (8,449), -543 (9,421)	+, -, -
***S100A10***	8	-4729 (8,687), -4728 (9,828), -4050 (8,178), -3913 (10,807), -3912 (9,350), -2303 (9,666), -2272 (8,449), -2271 (9,431)	+, -, +, +, -, -, +, -
***GSR***	6	-3066 (9,667), -3065 (8,199), -2431 (9,726), -2043 (8,017), -905 (10,023), -904 (8,229)	+, -, -, -, +, -
***ST8SIA1***	3	-3532 (11,197), -3531 (8,039), -2001 (8,841)	+, -, +
***CUL5***	3	-4798 (8,960), -643 (8,504), -642 (8,754)	+, +, -
***SCAP***	2	-3904 (8,449), -3903 (9,431)	+, -
***AKR1B1***	5	-3654 (8,372), -3594 (8,820), -3429 (9,409), -2950 (8,229), -2636 (8,960)	-, +, -, -, +
***ATP1B1***	6	-4514 (11,479), -4513 (10,785), -4151 (9,902), -2867 (9,095), -1545 (8,252), -1136 (8,960)	+, -, -, +, +, -
***VCAN***	2	-1256 (9,350), -224 (8,055)	+, -
***TRIM21***	7	-3372 (10,470), -3371 (9,706), -2169 (9,118), -2043 (8,322), -407 (8,055), -207 (8,515), -206 (10,627)	+, -, +, +, +, +, -
***GLRX***	7	-2391 (9,431), -2390 (8,449), -1766 (8,687), -1765 (8,566), -1108 (9,095), -1107 (8,485), -115 (8,213)	+, -, +, -, +, -, -
***SLC23A1***	3	-4955 (8,047), -3370 (12,419), -2782 (8,372)	+, -, +
***RPL29***	7	-4179 (8,147), -2366 (8,372), -703 (9,552), -702 (8,890), -624 (9,277), -593 (8,449), -592 (9,431)	-, -, +, -, -, +, -
***PSMB5***	6	-3113 (8,449), -3112 (9,431), -1295 (9,431), -1294 (8,449), -1105 (9,431), -1104 (8,449)	+, -, +, -, +, -
***ASNS***	3	-4092 (9,277), -2283 (9,104), -2087 (10,491)	+, -, +
***LDHB***	4	-4479 (9,491), -830 (9,902), -563 (9,431), -562 (8,449)	-, +, +, -
***RAD23A***	5	-2525 (8,449), -2524 (9,431), -156 (9,095), -155 (11,278), -37 (8,607)	+, -, +, -, -
***INSIG1***	5	-4207 (8,252), -3881 (8,259), -3535 (8,687), -2754 (9,421), -161 (8,754)	+, -, -, +, -
***PABPCP5***	3	-1789 (8,449), -1788 (9,431), -454 (9,341)	+, -, +
***ATOX1***	9	-4359 (8,199), -4358 (11,356), -2830 (9,134), -2656 (9,706), -2618 (8,017), -2313 (9,431), -2312 (8,449), -947 (9,949), -121 (9,956)	+, -, +, +, +, +, -, -
***PRKCSH***	9	-4688 (8,372), -4621 (9,491), -3737 (9,431), -3736 (8,449), -2854 (10,889), -2765 (8,298), -2764 (10,092), -121 (8,830), -120 (11,519)	-, -, +, -, +, +, -, +, -
***ISG15_***	5	-3964 (9,431), -3963 (8,449), -1932 (10,627), -1931 (12,578), -1603 (8,218)	+, -, +, -, -
***EEF2***	6	-3734 (10,572), -3278 (9,552), -3277 (8,890), -2791 (9,431), -2790 (8,449), -1565 (8,515)	-, +, -, +, -, -
***PCDH17***	1	-501 (8,199)	-
***TIMM17A***	11	-3771 (8,047), -3657 (8,952), -2621 (9,431), -2620 (8,449), -2157 (8,923), -1445 (9,431), -1444 (9,552), -1084 (9,431), -623 (9,431), -622 (8,449), -170 (8,047)	+, +, +, -, +, +, -, +, +, -, +
***RRAS***	4	-4729 (8,055), -3576 (9,104), -3575 (8,441), -230 (10,403)	-, +, -, +
***SYNCRIP***	4	-4703 (7.977), -1609 (10,480), -1608 (12,721), -77 (8,607)	+, +, -, -
***RPS10***	2	-3402 (7,980), -1980 (7,980)	+, -
***UBB***	4	-4351 (9,431), -4350 (8,449), -1719 (8,305), -1268 (9,350)	+, -, -, +
***RPSA***	**No binding sites detected**		
***PRMT1***	6	-4832 (8,305), -4831 (8,372), -4430 (8,441), -1711 (8,393), -763 (10,153), -762 (9,706)	+, -, +, -, +, -
***LMAN1***	1	-511 (8,444)	-
***YBX1***	6	-3039 (8,464), -2000 (9,585), -1590 (8,960), -1589 (9,277), -848 (11,455), -847 (10,755)	-, +, +, -, +, -

**Figure 3:**
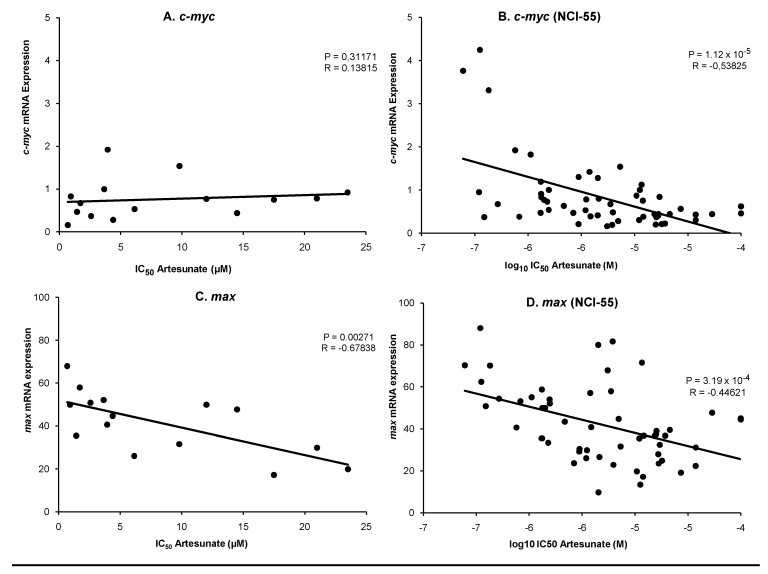
Linear regression of IC_50_ values for ART and mRNA expression of c-Myc **(**A) and Max (C) in our cell lines and linear regression of log_10_ IC_50_ values for ART and mRNA expression of c-Myc (B) and Max (D) in the NCI cell line panel. Significance levels were calculated using Pearson’s correlation test.

Since the transcriptional regulation of c-Myc is activated by binding of c-Myc to its dimerization partner, max, we correlated the microarray-based mRNA expression of c-Myc and Max, with the IC_50_ values for ART of our panel of cell lines in comparison to the cell line panel of the National Cancer Institute (NCI), USA. As shown in Figure 3A, there was no significant correlation between IC_50_ values for ART and c-Myc mRNA expression in our cell line panel (*P* = 0.3117, *R* = 0.13815). In contrast, in the NCI cell line panel there was a significant inverse correlation (*P* = 1.12 x 10^-5^, *R* = -0.53825) between IC_50_ values for ART and c-Myc mRNA expression (Figure 3B). Interestingly, we observed an inverse correlation between max mRNA expression and IC_50_ values for ART in both cell line panels (our cell line: *P* = 0.00271, *R* = -0.67838; NCI cell line: *P* = 3.19 x 10^-4^, *R* = -0.44621) (Figure 3C and D).

## 3. Discussion

### 3.1. Identification of candidate genes by microarray and COMPARE analyses

In the present investigation, we analyzed molecular determinants of response of tumor cell lines towards ART by means of microarray-based mRNA expression profiling and correlation of the IC_50_ values for ART of 39 tumor cell lines with the mRNA expression levels of these cell lines [16,17] by COMPARE analysis. This approach has previously been applied to unravel the mode of action of novel compounds [18,19,20]. Cluster and COMPARE analyses are also useful for comparing gene expression proﬁles with IC_50_ values for investigational drugs to identify candidate genes for drug resistance [21] and to identify prognostic expression proﬁles in clinical oncology [22].

We identified genes from diverse functional groups such as structural constituents of ribosomes (*RPL29*), ATP-binding cassette (ABC) transporters (*ABCC3*), kinases (*PRKCSH, ITPK1, IKBKG*, *DDR2*), cellular antioxidant defense and carcinogenesis (*ATOX1*), organization of actin cytoskeleton (*RRAS*), regulation of carcinogenesis (*SMAD3*, *WNT7A*), cell adhesion and growth of malignant cells (*ST8SIA1*), apoptosis and cell proliferation (*CSE1L*), cell cycle progression and differentiation (*S100A10*) and metastasis (*HMGA1*, *RPSA*). Next, we classified those genes from the COMPARE analyses into genes correlating with sensitivity or resistance towards ART. *ABCC3, ITPK1, IKBKG, SMAD3, WNT7A, ST8SIA1, S100A10, AKR1B1, AKR1C1* and *ALDH3A1* were identified as genes correlating with ART resistance. Sensitivity genes for ART were *ATOX1*, *RPL29*, *PRKCSH*, *DDR2*, *RRAS*, *CSE1L*, *HMGA1* and *RPSA*. None of these genes have yet been reported to play a role for cellular response towards ART. Some of them, however, are involved in resistance to other drugs. This might also explain their role for response to ART. 

Ribosomal proteins represent a classical mechanism of resistance towards antibiotics. Streptomycin resistance is based on the modification of an asparatic acid moiety in the ribosomal protein S12 [23]. *RPL29* encodes a cytoplasmic ribosomal protein that is a component of the 60S subunit, and is also a peripheral membrane protein expressed on the cell surface that directly binds heparin. There is no evidence in the literature yet that cancer drug resistance is correlated with *RPL29*.

Multidrug resistance (MDR) is based on multiple mechanisms, one of which is drug efflux by ATP-binding cassette (ABC) transporters. They are involved in the active transport of phospholipids, ions, peptides, steroids, polysaccharides, amino acids, bile acids, pharmaceutical drugs and other xenobiotic compounds [24]. In humans, 49 different ABC transporters have been identified, which are classified into seven sub-families (A–G) [25]. *ABCB1* (P-glycoprotein, P-gp, MDR1), *ABCC1*-*C6* (MRP1-6) and *ABCG2* (BCRP) confer resistance to cytostatic drugs of tumors and contribute to the failure of tumor chemotherapy [26]. With our pharmacogenomic approach, we found that high *ABCC3* expression was associated with resistance towards ART. *ABCC3* belongs to the ABC transporters and encodes multidrug resistance-associated protein 3 (MRP3). Over-expression of *ABCC3* results in high-level resistance to methotrexate [27]. Previously, we showed that ART is neither a substrate for *ABCB1* and *ABCG2* [28], nor an inhibitor of both ABC transporters [29]. It can be speculated that *ABCC3* might also affect susceptibility of cancer cells towards ART.

Protein kinases (PK) are fundamental for many cellular processes such as proliferation, apoptosis and differentiation [30]. Further, PKs are also involved in signal transduction related to resistance towards established anticancer drugs [31]. We previously reported that EGFR and its downstream kinases confer resistance towards ART [28,32]. In the present study, we identified four novel genes coding for kinases, which were correlated to response of tumor cells towards ART.

Copper is essential, although extremely toxic in high concentrations, intracellular copper concentrations are tightly controlled. Within the cell, copper is distributed by metallochaperones, including the small cytoplasmic protein *ATOX1* [33]. *ATOX1* bas been implicated in resistance to cisplatin [34]. Likewise, binding of ART to *ATOX1* might induce ART resistance.

The Y-Box-binding protein-1 (*YBX1*), a member of the cold-shock domain DNA- and RNA-binding protein superfamily, is known to mediate drug resistance. *YBX1* is known to be involved in DNA repair, transcription, splicing, translation, and confers cisplatin resistance in several cancers [35]. *YBX1* expression has been shown to have clinicopathological significance to be a potential predictive marker of recurrence in nasopharyngeal cancer patients [36]. Moreover, *YBX1* regulates the expression of the multidrug-resistance gene *MDR1/ABCB1*, which encodes P-glycoprotein [37,38]. Over-expression of P-gp is associated with MDR by reducing drug accumulation in resistant cells [39]. In breast cancer, *YBX1* expression is discussed to be a potential marker of drug resistance and could possibly aid in selection of the appropriate adjuvant chemotherapy regime for breast cancers [40]. For NSCLC, nuclear *YBX1* expression is an independent prognostic marker [37,38].

*AKR1B1* encodes one of three daunorubicin reductases, namely aldose reductase. Over-expression of *AKR1B1* results in drug resistance towards daunorubicin [41]. *AKR1C1* is a member of the aldo-keto reductase superfamily that is involved in the activation of carcinogenic polycyclic aromatic hydrocarbons [42]. Interestingly, transfection of *AKR1C1* in NSCLC cells contributes to drug resistance towards cisplatin and adriamycin *via* the activation of PKC. *AKR1C1*-associated drug resistance may be related to altered control of DNA repair and apoptosis [43].

*ALDH3A1* encodes an antioxidant enzyme (aldehyde dehydrogenase) with several postulated protective roles that include detoxification of peroxidic aldehydes and scavenging of free radicals. Its expression has been implicated in clinical resistance to cyclophosphamide [44]. In addition, *ALDH3A1* contributes to the multidrug resistance-conferring function of the metastasis gene metadherin [45]. These genes could provide new targets for overcoming ART resistance, although underlying mechanisms remain to be elucidated.

### 3.2. Signaling pathway analyses

To analyze whether the genes identified belong to certain signal transduction pathways, which may be relevant for mediating the cytotoxic effects of ART, we applied Ingenuity Pathway Analysis and found that TNF- and TP53- signaling pathways were affected. 

The tumor necrosis factor (TNF) is a cytokine that is able to induce apoptotic cell death and to inhibit tumorigenesis. Also, TNF induces nuclear factor 'kappa-light-chain-enhancer' of activated B-cells (NF-κB) activation [46]. Considering the fact, that chemotherapeutic agents also have the ability to induce NF-κB activation through the up-regulation of anti-apoptotic gene products that leads to drug resistance [47], we conclude that TNF might play a role in ART resistance.

Since the early 1990s, the *TP53* tumor suppressor gene has been suggested to be involved in drug resistance [48]. Studies over the past decade have provided stringent evidence linking mutations in TP53 with drug resistance to the anthracyclines but also to mitomycin in hematological malignancies and breast cancer [49]. In the present investigation, we showed that TNF- and TP53-signaling pathways might also be relevant for ART response rates of tumor cells.

We performed signaling pathway analysis to compute biological functions that are most probably affected by ART. Interestingly, lipid metabolism and drug metabolism were the second significantly important biological functions. Lipids are involved in the formation of biological membranes and the lipid composition of vesicles involved in membrane traffic [50]. Vesicular drug binding, processing and extrusion was hypothesized as a general mechanism involved in drug resistance [51]. Formation of membrane vesicles was proven for mitoxantrone resistance [52]. Based on our result, membrane vesicles for extrusion and exocytosis of chemotherapeutics might be one mechanism of ART resistance. 

By signaling pathway analysis, we also determined potential mechanisms of toxicity, *e.g.* xenobiotic metabolism, oxidative stress, TGF-β signaling and PXR/RXR activation. Epidermal growth factor (EGF) and its receptor (EGFR) as well as the EGFR-coupled Ras > Raf > MEK > ERK pathway are known to affect the survival of cancer cells upon therapeutic treatment. Indeed, we observed that the Ras > Raf > MEK > ERK pathway is an important signaling route for ART resistance [32]. Another downstream gene of EGFR, e.g., phosphatidylinositol 3'-kinase (PI3K)/AKT has been shown to contribute to cisplatin resistance by promoting cell proliferation and increasing drug metabolism and resistance to apoptosis [53,54]. In addition, EGFR differentially modulates the PI3K/AKT pathway in regulating tumor cell growth and dormancy [55]. Also, transforming growth factor-beta1 (TGF-β1) plays an important role in drug resistance induced by glucocorticoids in ovarian cancer cells [56]. Cooperative signaling between EGFR and TGF-β regulates tumor invasion, neo-angiogenesis and inflammation [57]. As signaling is an extension of EGFR, a contribution of TGF-β1 in EGFR-related ART resistance can be hypothesized. 

The pregnane X receptor (PXR)/retinoid X receptor (RXR) heterodimer regulates a constellation of genes involved in xenobiotic detoxification, the cytochrome P450 (CYP) [58]. In particular, CYP3A is involved in the hepatic detoxification of more than 50% of prescription drugs [59]. As ART is a xenobiotic, PXR/RXR has to be taken into account as a mechanism of detoxification.

Among the pleiotropy of canonical pathways that might be affected by ART, five top pathways were identified by signaling pathway profiling: lipopolysaccharides (LPS)-stimulated MAPK signaling, apoptosis signaling, PI3K/AKT signaling, pyruvate metabolism, and angiopoietin signaling. 

LPS activates the inducible nitric oxide synthase (iNOS) expression pathway and in this context, NO generation and signaling play a role in exhibiting cytotoxic activity of ART [60]. This is in concordance with the finding of the present study, that ART might affect LPS-stimulated MAPK signaling. 

Research on apoptosis has increased substantially since the early 1990s. In the past, we showed that ART induced apoptosis in KG-1a leukemia cells [8], and that a number of apoptosis-regulating genes (*LOC51272, CIDEB, PDCD2, BAG1, BAG3, MADD*) correlated with cellular responses towards ART [28]. The present study reveals *CSE1L* as apoptosis-regulating gene associated with cellular response towards ART. In an earlier study, we analyzed the role of ferrous iron in the cytotoxicity of artemisinins toward tumor cells. ART and ART plus ferrous iron induced apoptosis in CCRF-CEM cells [61]. Apoptosis by ART is mainly induced by the mitochondrial pathway *via* generation of reactive oxygen species (ROS) in leukemic T-cell lines, a mechanism different from doxorubicin [6]. The active moiety of ART is an endoperoxide bridge that generates carbon-centered free radicals and oxidative stress upon cleavage. Oxidative stress appears to be necessary for the antimalarial activity of ART and suggests that antioxidant defenses act in combination to affect the cellular response to ART [11]. Indeed, in the present study we observed correlations of oxidative stress and mitochondrial dysfunction to ART. This aspect still needs detailed elucidation in future. 

Pyruvate supplies energy to cells in the citric acid cycle, and can also be converted to carbohydrates via gluconeogenesis, to fatty acids or energy through acetyl-CoA, to the amino acid alanine and to ethanol [62]. High concentration of pyruvate inhibits the activity of pyruvate dehydrogenase kinase (PDK) 1, -2, and -4, but not PDK3 [63]. Induction of PDK3 by hypoxia blocks the conversion of pyruvate to acetyl-CoA and reduces oxygen consumption. PDK3 may represent the critical molecule that controls the metabolic switch of cancer cells from oxidative phosphorylation to aerobic glycolysis (Warburg effect) [63]. Based on our present data, ART-induced over-expression of PDK3 may be a new mode of anti-cancer activity of ART.

Angiopoietin signaling was correlated with following genes: *IKBKG, RRAS2, RRAS*. *IKBKG* belongs to one of our identified four novel genes coding for kinases, which were correlated to response of tumor cells towards ART. *RRAS* regulates the organization of the actin cytoskeleton. Angiopoietin-induced activation during tumor growth, development, and metastasis suggests that the ability to modulate the receptor-ligand interactions would have onco-therapeutical applications. In previous studies, we and others confirmed the anti-angiogenical effect of ART and its metabolite dihydroartemisinine [64]. Inhibition of angiogenesis may be a general mechanism of artemisinin derivatives to inhibit tumor growth *in vivo* [65]. This is in accordance to the present correlation of angiopoietin signaling with ART.

### 3.3. Transcription factor downstream gene analyses

It was a striking and surprising result that a considerable number of genes identified by microarray and COMPARE analyses are not linked to cancer biology, e.g. genes involved in the survival of cochlear outer and inner hair cells (*SLC12A7*), the active cellular uptake of carnitine (*SLC22A5*), regulation of sterol biosynthesis (*SCAP*), the synthesis of asparagines (*ASNS*) and regulation of gene expression (*UBB*), do not have obvious links to tumor diseases. This poses the question, whether other mechanisms than activation of cancer-specific signaling pathways may contribute to mRNA expression profiles. We hypothesized that transcription factors may transcriptionally activate downstream genes at least partially independent of whether they belong to certain signaling routes. Hence, transcription factors active in ART-sensitive tumor cells regulate not only genes affecting ART sensitivity, but also genes unrelated to cellular response towards ART. This may explain, why we found many genes without obvious connection to cancer or drug response. Since c-Myc is known to affect downstream processes in tumor cells [16] and we previously reported that the expression of c-Myc is correlated with sensitivity of cancer cells to ART [28], we took c-Myc as an example to further investigate this hypothesis. Therefore, we performed a gene promoter analysis of these genes, which were significantly correlated in our cell line panel with response to ART.

Indeed, the vast majority of genes contained one or more binding motifs in their promoters for c-Myc. This result is conceivable with the fact that the expression of c-Myc and its dimerization partner, Max, significantly correlated with response of tumor cells to ART. Our explanation of these results is that the transcriptional regulation of downstream genes by c-Myc/Max determines cellular response towards ART. The c-Myc/Max-regulated genes may in part affect ART sensitivity. Another fraction of c-Myc-Max regulated genes might not causally be linked with cellular response to ART, but are differentially expressed in ART-sensitive and ART-resistant tumors. Another possibility is that the therapeutic potential of artemisinin-type drugs is much larger than malaria and cancer. Therefore, the molecular architecture of cells might be exploited to treat also other diseases by ART.

The gene with the highest potential binding sites for c-Myc, *KRT4*, belongs to the ART resistance genes and encodes a protein of the keratin gene family. The type II cytokeratins consist of basic or neutral proteins, which are arranged in pairs of heterotypic keratin chains co-expressed during differentiation of simple and stratified epithelial tissues. This type II cytokeratin is specifically expressed in differentiated layers of the mucosal and esophageal epithelia with family member KRT13. Mutations in these genes have been associated with White Sponge Nevus, characterized by oral, esophageal, and anal leukoplakia.

The gene with the second highest potential binding sites, *SLC12A7*, also belongs to the ART resistance genes. It encodes a potassium-chloride transporter, and has second highest number of potential binding sites for c-Myc. It mediates electroneutral potassium-chloride co-transport when activated by cell swelling. It may mediate K^+^ uptake into Deiters' cells in the cochlea and contribute to K^+^ recycling in the inner ear. It is important for the survival of cochlear outer and inner hair cells and the maintenance of the organ of Corti. In addition, it may be required for basolateral Cl^-^ extrusion in the kidney and contribute to renal acidification.

Another gene associated with resistance towards ART, *S100A10,* has eight potential binding sites for c-Myc. It encodes a member of the S100 family of proteins. S100 proteins are localized in the cytoplasm and/or nucleus of a wide range of cells, and are involved in the regulation of a number of cellular processes such as cell cycle progression and differentiation. This protein may function in exocytosis and endocytosis. Because *S100A10* induces the dimerization of ANXA2/p36, it may function as a regulator of protein phosphorylation in that the ANXA2 monomer is the preferred target of tyrosine-specific kinases.

We also found genes associated with sensitivity towards ART with high numbers of binding sites for c-Myc, e.g. *TIMM17A, ATOX1*, *TIMM17A, PRKCSH*, *RPL29* and *YBX1. TIMM17A* encodes trans-locase of inner mitochondrial membrane 17. It is an essential component of the TIM23 complex, a complex that mediates the translocation of transit peptide-containing proteins across the mitochondrial inner membrane. *ATOX1* encodes a copper chaperone that plays a role in copper homeostasis by binding and transporting cytosolic copper to ATPase proteins in the trans-Golgi network for subsequent incorporation into ceruloplasmin. This protein also functions as an antioxidant against superoxide and hydrogen peroxide, and therefore, may play a significant role in cancer carcinogenesis and may be important in cellular antioxidant defense. *PRKCSH* encodes the beta-subunit of glucosidase II, an N-linked glycan-processing enzyme in the endoplasmic reticulum. This acidic phosphoprotein is known to be a substrate for protein kinase C. Mutations in this gene have been associated with the autosomal dominant polycystic liver disease. *RPL29* encodes a cytoplasmic ribosomal protein that is a component of the 60S subunit. The protein belongs to the L29E family of ribosomal proteins. The protein is also a peripheral membrane protein expressed on the cell surface that directly binds heparin. *YBX1* binds to splice sites in pre-mRNA and regulates splice site selection. It contributes to the regulation of translation by modulating the interaction between the mRNA and eukaryotic initiation factors (By similarity). Moreover, it binds to promoters that contain a Y-box (5'-CTGATTGGCCAA-3'), such as HLA class II genes. *YBX1* regulates the transcription of numerous genes and promotes separation of DNA strands that contain mismatches or are modified by cisplatin. Also, it has endonucleolytic activity and can introduce nicks or breaks into double-stranded DNA and therefore may play a role in DNA repair.

## 4. Experimental

### 4.1. Drugs and reagents

ART was obtained from Saokim Co. Ltd. (Hanoi, Vietnam). Dimethylsulfoxide (DMSO; Sigma) was used to dissolve and prepare stock solutions of ART (20 mg/mL). 

### 4.2. Cell lines

The panel of cell lines for the present investigations consisted of 39 human tumor cell lines representing non-small cell lung cancer (A549, NIH-H23, NIH-460, WT-E, BH-E, SKLC-6, QU-DB), colon cancer (HT29, COLO205, WiDr, LoVo, DLD-1, HCT116, SW620, HCT15, BE, SW480, HCG2998, KM12) and ovarian carcinoma (OVCAR2, OVCAR3, OVCAR4, OVCAR5, OVCAR8, OVCAR10, 2008, C13, C30, C200, SKOV3, OAW42, UPN251, PEO1, PEO4, CP70, A1847, A2780, A2780-AD10, A2780-TX) [66]. Cell lines wree obtained from American Type Culture Collection (ATCC; Manassas, VA, USA) and used in passages 60-100. The human ovarian cancer cell lines contained highly cisplatin resistant cell lines of the A2780/C-series (C30 and C200) [67]. Furthermore, adriamycin A2780-AD10, and paclitaxel-resistant cells (A2780-TX) were used [67].

Cells were maintained at 37 °C in a humidified incubator containing 5% CO_2_ in RPMI-1640 medium (Life Technologies, Grand Island, NY, USA) supplemented with 10% (vv^-1^) fetal calf serum (Atlanta Biologicals, Atlanta, GA, USA), 100 μg mL^-1^ streptomycin, 100 U mL^-1^ penicillin, 0.3 mg mL^-1^ glutamine and 0.25 U mL^-1^ insulin (porcine).

### 4.3. MTT assay

Cytotoxicity in terms of IC_50_ values for the four platinum drugs were determined using the 3-(4,5-dimethyl-thizol-2-yl)-2,5-diphenyltetrazolium bromide (MTT) assay [68]. Cells (100 – 4,000/well) were plated in 150 μL of medium/well in 96-well microtiter plates. Following overnight incubation, cells were exposed to a range of drug concentrations. After 72 h, 40 μL of 5 mg mL^-1 ^MTT were added per well and the plates were incubated for 2 h at 37 °C. The cells were then lysed by adding 100 μL of 20% (wv) sodium dodecyl sulphate (SDS), 50% (vv) *N,N*- dimethylformamide (pH 4.7), and then incubating overnight at room temperature. The absorbance at 595 nm was determined using a Bio-Tek EL_X_800 microplate reader (Bio-Tek Instruments, Winooski, VT, USA). The reported values are the average of triplicate determinations made on at least two separate occasions. 

### 4.4. RNA isolation

Total cellular RNA was extracted from the cell lines by a modification of the single-step method [69,70] using guanidine isothiocyanate. RNA was precipitated from the aqueous phase by the addition of isopropanol. Following centrifugation, RNA pellets were washed with 75% ethanol, resuspended in DEPC-treated water and treated with DNase I. RNA integrity was assessed by ethidium bromide staining following agarose-gel electrophoresis and quantified by absorbance at 260 nm. Samples were stored at -80 °C under ethanol.

### 4.5. Preparation of cDNA probes

Total RNA (2.0 μg) was combined in a 1.5 mL microcentrifuge tube with 10 μg of oligo-dT (Research Genetics) in a final volume of 10 μL and incubated at 70 °C for 10 min and placed on ice for 5 min. The cDNA synthesis reaction contained a mixture of 6.0 μL of 5 × First Strand Buffer (Life Technologies), 1.0 μL 100 mΜ DTT, 1.5 μL dA/dG/dT (10 mM each), 10 μL [a^-33^P] dCTP (100 μCi), and 1.5 μL MMLV reverse transcriptase (200 U μL^-1^). Samples were incubated at 37 °C for 1.5 h. Nucleotides and unincorporated ^33^P were removed by gel chromatography using Micro Bio-Spin 30 columns (Bio-Rad Laboratories, Hercules, CA, USA). The samples were denatured by incubating at 100 °C for 3 min and placed on ice.

### 4.6. cDNA microarray screening

Microarray analysis was carried out using the recommended procedure supplied by Research Genetics Inc. The Named Human Genes filters (GF211), which are spotted with 4132 cDNA elements, were pre-hybridized in 5 mL MicroHyb solution (Research Genetics) containing 5 μg of poly-dA overnight at 42 °C in a hybridization oven (Model H010-1, Stovall Life Science Inc., Greensboro, NC, USA). The denatured probes were then added to this solution and incubated 14–18 h at 42 °C. The membranes were washed briefly with 200 mL of wash buffer I (2×SSC, 1% SDS) at room temperature, followed by two 20 min washes with the same buffer at 50 °C, and two washes with 200 mL of wash buffer II (0.5 × SSC, 1% SDS) at 55 °C for 20 min. The filters were removed, immediately wrapped in plastic and imaged on a Storm 840 phosphorimager (Molecular Dynamics, Sunnyvale, CA, USA), following a 5-day exposure to the imaging plate. For reuse, membranes were stripped by incubating for 20 min in 0.5% (wv) SDS that had been brought to 100 °C and repeating. Stripped filters were re-imaged following a 24 h exposure to insure complete removal of radioactivity.

### 4.7. Data analyses

The data obtained from the phosphorimager was analyzed using Pathways software (Research Genetics). This software utilizes the raw image obtained from the phosphorimager in order to align the image and determine expression values for all of the elements. The raw data were downloaded into Microsoft Excel for final analysis. Also, a signal-to-noise ratio was obtained for all of the hybridized filters by dividing the mean of the raw data by the background value. Only the hybridizations that yielded signal-to-noise ratios above 1.5 were used in the subsequent data analysis. In order to correct for background, the average value of the lowest 1% of the genes expressed for each sample was subtracted from each raw data point to obtain a background corrected expression value. The data were then normalized by subtracting the mean and dividing by the standard deviation of the expression values for the entire data set for each cell line (global means method). RNA from each cell line was subject to microarray analysis at least twice. The normalized data obtained from duplicated hybridizations were averaged to obtain a final data set. Reproducibility of the data was assessed by calculating a percent error (S.D./mean × 100) for each gene element.

The data were cropped to a final set of 2,000 elements by eliminating genes with relatively low expression and low standard deviation across the panel of cell lines. This was carried out by exemplarity measuring gene expression by quantitative ‘real-time’ PCR (qRT-PCR). We considered a gene ‘validatable’ if the Spearman and/or Pearson correlation coefficient was ≥0.6 for the microarray/PCR data. A factor (P) was calculated by multiplying the average of the expression of each gene for all the cell lines with the standard deviation. Genes with P-values below 100,000 could not be consistently validated by RTq-PCR and were eliminated. We also eliminated genes in which the median expression value across the cell lines was low (see Web Supplement for more detail).

The identification of candidate drug sensitivity/resistance genes was carried out by several statistical methods. Correlation coefficients (Pearson) were calculated for the expression of each gene relative to artesunate sensitivity for the entire panel of cell lines. The correlation coefficients were then ranked from highest to lowest and *vice versa* in order to obtain a list of genes associated with proliferation and/or platinum drug sensitivity. Information about genes was obtained by the web-based database GeneCards^®^ that provides concise genomic, proteomic, transcriptomic and functional information on all known and predicted human genes (http://www.genecards.org/index.shtmL/).

### 4.8. Statistical analyses

COMPARE analyses were performed with software implemented into the web site of the NCI (http://dtp.nci.nih.gov). COMPARE analyses yielded rank-ordered lists of compounds. Every compound of the Standard Agent Database of the NCI was ranked for similarity between its modulation of *in vitro* cell growth patterns and the modulation of *in vitro* cell growth patterns of a selected seed or probe compound [71]. To obtain COMPARE rankings, a scaler index of similarity between the seed compound cell growth pattern and the pattern for each of the COMPARE database compounds was created. This methodology has previously been exploited to identify the presumable mode of action of investigational drugs by comparing their IC_50_ profiles of the NCI cell lines with those of drugs with well-established mechanisms of action [19]. Pearson’s correlation test determined the correlation of rank positions of values. Ordinal or metric scaling of data is suited for the test and transformed into rank positions. There is no condition regarding normal distribution of the data set for the performance of this test. This test was implemented into the WinSTAT Program (Kalmia Co., Cambridge, MA, USA).

### 4.9. Identification of signaling pathways

The Ingenuity Pathway Analysis software (IPA) (Ingenuity Systems, Mountain View, CA, USA; www.ingenuity.com) was utilized to identify networks and pathways of interacting genes and other functional groups in genomic data. Using the IPA Functional Analysis tool, we were able to associate biological functions and diseases to the experimental results. Moreover, we used a biomarker filter tool and the Network Explorer for visualizing molecular relationships. 

### 4.10. Binding motif analyses in gene promoters

The ConSite program was used to detect binding sites of the transcription factor c-Myc in gene promoter sequences of the determined microarray analyses-based genes. ConSite is available at (http://www.phylofoot.org/consite/).

## 5. Conclusions

The application of microarray-based mRNA profiling and COMPARE analysis led to the identification of genes, whose expression was associated either with high IC_50_ values or low IC_50_ values for ART. Therefore, these genes may determine resistance or sensitivity of tumor cells towards ART. This viewpoint is conceivable for genes involved in ribosomal activity, drug transport, cellular antioxidant defense, apoptosis, cell proliferation, cell cycle progression *etc*. An investigation of underlying signal transduction pathways by the Ingenuity Pathway Analysis software suggested a role of the signaling pathways related to TNF and the tumor suppressor p53. On the other hand, there were genes without obvious functional link to cellular response to ART, such as genes either involved in the survival of cochlear outer and inner hair cells *etc*. We proved the hypothesis that ART might influence the activity of transcription factors, which regulate downstream genes involved or not involved in response of cancer cells towards ART. This would explain the identification of genes with and without obvious relation to the cytotoxic activity of ART by microarray and COMPARE analyses. Investigating the binding motifs for the transcription factors c-Myc and max we indeed found that 53 of 56 genes contained one or more binding sites for c-Myc/Max in their promoter sequences. We conclude that c-Myc and Max-mediated transcriptional control of gene expression might contribute to the therapeutic effects of ART in cancer cells, but may also confer unwanted side effects by affecting therapy-unrelated genes.
